# MOCA-Net: A Model for Automatic Segmentation of Retrogressive Thaw Slumps from Sentinel-2 Imagery Along the Qinghai–Tibet Engineering Corridor

**DOI:** 10.3390/s26103267

**Published:** 2026-05-21

**Authors:** Yijiang Li, Qiong Li, Guoxin Chen, Wenqi Li, Changyan Bao

**Affiliations:** 1 School of Civil Engineering and Water Resources, Qinghai University, Xining 810016, China; qtqt12321@126.com (Y.L.); liwenqi2456@163.com (W.L.); 2 Key Laboratory of Water Ecology Remediation and Protection at Headwater Regions of Big Rivers, Ministry of Water Resources, Xining 810016, China; chenguoxin@qhu.edu.cn; 3 Laboratory of Ecological Protection and High Quality Development in the Upper Yellow River, Qinghai University, Xining 810016, China; 4 School of Geological Engineering, Qinghai University, Xining 810016, China; 5 State Key Laboratory of Plateau Ecology and Agriculture, Qinghai University, Xining 810016, China; 6 Qinghai Remote Sensing Center for Natural Resources, Xining 810010, China; bcq646282@163.com

**Keywords:** retrogressive thaw slump, deep learning, semantic segmentation, MOCA-Net, Sentinel-2

## Abstract

**Highlights:**

**What are the main findings?**

- We developed MOCA-Net, a Transformer-based deep learning framework specifically designed for RTS segmentation to address the challenges of complex morphology and fuzzy boundaries in medium-resolution satellite imagery.
- MOCA-Net outperformed seven mainstream baseline models, achieving a mean IoU of 0.8609 and an RTS-class IoU of 0.7473, with improved performance in capturing accurate morphologies and boundaries.

**What are the implications of the main findings?**

- The proposed model unlocks the capability of Sentinel-2 imagery for accurate RTS segmentation, making it promising for applications over large spatiotemporal extents.
- This accurate automatic segmentation is of great value for climate change research and infrastructure risk assessment along the Qinghai–Tibet Engineering Corridor (QTEC).

**Abstract:**

Retrogressive thaw slumps (RTSs) serve as key indicators of global climate change and also pose significant risks to critical infrastructure along the Qinghai–Tibet Engineering Corridor (QTEC). Accurate automatic segmentation of RTSs using Sentinel-2 imagery is of great value for climate change research and risk assessment, owing to the dataset’s ready availability and extensive spatiotemporal coverage. However, this segmentation task remains challenging due to the complex morphology and variable sizes of RTSs, as well as their low contrast and fuzzy boundaries against the surrounding landscape in medium-resolution satellite imagery. To deal with these challenges, this study proposes the Multi-Scale Object-aware Context Attention Network (MOCA-Net), which enhances the Swin Transformer backbone through two critical components: the Feature Enhancement Network and Enhanced Decoder. Evaluation metrics show that MOCA-Net outperforms seven mainstream baseline models, achieving a Mean Intersection over Union (mIoU) of 0.8609 and an RTS-class IoU of 0.7473. The qualitative visual evaluation further confirms MOCA-Net’s improved performance in delineating RTSs through more accurate morphologies and boundaries. Ablation studies confirm that each designed component contributes to the MOCA-Net’s segmentation performance, and their combination yields more balanced results. This model unlocks the capability of Sentinel-2 imagery for accurate RTS segmentation, making it promising for applications over large spatiotemporal extents.

## 1. Introduction

The Qinghai–Tibet Plateau (QTP) has the world’s highest and largest permafrost region in low- and middle-latitude regions [1,2]. The permafrost on the QTP is warmer than the high Arctic [3] and nearly 40% of permafrost is considered to be especially warm and unstable (ground temperature > −0.5 °C) [4,5]. Warm permafrost which is more sensitive to climate warming can lead to higher degradation potential [6,7,8]. Thaw-induced slope failure plays a critical role in the permafrost degradation process, posing considerable threat to infrastructure in cold regions and having a significant environmental impact [7,9,10,11,12]. Retrogressive thaw slumps (RTSs) are a kind of typical and regionally widespread slope failure that occurs in the permafrost region, and are triggered by the thaw of permafrost and the melt of massive ground ice [13].

The Qinghai–Tibet Railway, the Qinghai–Tibet Highway and other linear engineering facilities (e.g., Qinghai–Tibet DC power transmission line) are all located along the central Qinghai–Tibet Engineering Corridor (QTEC) from Golmud to Lhasa. RTSs threaten the stability of infrastructure on the QTP, modify landscapes, and accelerate the organic carbon emission [14,15,16,17]. Inventory maps of RTSs are important to evaluate risk and vulnerability [9,15], perform carbon cycle studies [16], and evaluate landslide events [18,19]. As a prerequisite for these applications, a robust and effective method for detecting and segmenting RTSs is essential.

Given that RTS is a type of landslide, the methods employed for landslide identification remain effective for RTSs. Traditional methods include image visual interpretation [20,21,22], object-based image analysis (OBIA) algorithms [23,24,25] and pixel-based classification [26,27,28,29]. Visual interpretation generally yields accurate results but is time-consuming and labor-intensive, making it difficult to create RTS inventory maps for large regions and long time series. The OBIA method is usually a two-step process including segmentation and classification [30,31]. Segmentation refers to grouping small pixels together into vector objects, and classification refers to identifying target objects based on several object characteristics from spectral, spatial, hierarchical, textural, and morphological information [31]. The effective object characteristics are all manually designed and heavily rely on the research experience in feature engineering [32,33,34]. Manual feature engineering in the OBIA method is also costly, exhaustive and time-consuming. The pixel-based methods, such as maximum likelihood, minimum distance, parallelepiped, ISODATA, and K-means, have been widely used for per-pixel classification and natural hazard inventory mapping, such as landslide detection [26,27,28]. However, these methods rely solely on the spectral information of individual pixels, ignoring geometric and contextual information [35,36,37], and fail to account for the intricate textures and significant spectral heterogeneity inherent in the imagery [38].

Machine learning offers a promising data-driven method for automatically identifying RTSs or landslides, such as decision trees, support vector machines, artificial neural networks, random forest, etc., [35,39]. Compared to traditional methods, machine learning demonstrates superior performance due to its enhanced capacity for automatically mining high-dimensional data [40]. However, these aforementioned algorithms are fundamentally pixel-based approaches, making them difficult to effectively capture complex spatial structural relationships. Additionally, their vulnerability to overfitting and dependence on manual feature engineering hinder the application of machine learning to complex tasks and large-scale data mining.

Alongside notable advances in algorithms, increases in computational power, and the availability of large-scale datasets, deep learning, as a key subfield of machine learning, has become a dominant approach for solving complex problems in fields such as computer vision, natural language processing, and beyond [41]. Given that the identification of RTSs represents a typical semantic or instance segmentation task in the field of computer vision, deep learning models designed for such tasks are inherently well suited and hold considerable potential for RTS mapping [34,36,42,43,44,45,46]. Recent studies have applied deep learning models to RTS mapping from remote sensing imagery, including CubeSat-based mapping, transfer learning strategies, and deep neural network frameworks [47,48,49,50], demonstrating the feasibility of automated RTS identification. However, although previous studies have demonstrated the feasibility of deep learning for RTS mapping, accurate delineation of RTSs from medium-resolution Sentinel-2 imagery still remains challenging. Compared with landslides, the surface area of RTSs is much smaller, ranging from 0.02 to 20 ha with an average of 1.44 ha [51]. In addition, the colors and spectral characteristics of RTSs are similar to bare land in alpine meadow areas and other types of ground collapse, making target–background discrimination and boundary delineation more difficult in medium-resolution imagery.

Despite the suitability of high-resolution (HR) and very-high-resolution (VHR) remote sensing imagery for creating RTS inventories [52], their utility remains limited due to the late development of these technologies and the lack of freely accessible data. Fortunately, the freely available Sentinel-2 satellite data offer a reliable alternative for its relatively high spatial resolution of 10 m, high global revisit frequency of 5 days, long temporal coverage of nearly 10 years, rich multispectral information, and high data quality [53,54,55]. Crucially, this extensive temporal archive provides invaluable data for characterizing the spatiotemporal evolution of RTSs, which is essential for understanding long-term permafrost degradation dynamics. Therefore, a robust model for automatic segmentation of RTSs from Sentinel-2 imagery is of great practical significance. However, RTS segmentation in Sentinel-2 imagery is hindered by the complex morphology and variable sizes of RTSs, as well as their low contrast and fuzzy boundaries against the surrounding landscape in medium-resolution data.

To address this challenge in RTS mapping from medium-resolution Sentinel-2 imagery, this study proposes a Multi-Scale Object-aware Context Attention Network (MOCA-Net), an end-to-end deep semantic segmentation framework. MOCA-Net utilizes the Swin Transformer as backbone for its powerful ability of hierarchical feature extraction. It employs a three-stage cascaded architecture comprising an encoder, a Feature Enhancement Network, and an Enhanced Decoder to improve target–background discrimination, contextual refinement, and boundary recovery.

The main contributions of this manuscript are as follows: (1) We develop MOCA-Net, a deep semantic segmentation framework featuring a three-stage cascaded architecture with dedicated feature enhancement and sophisticated decoding stages, specifically designed to improve target–background discrimination, contextual refinement, and boundary recovery for accurate RTS mapping from satellite imagery. (2) We evaluate the effectiveness of the MOCA-Net model in segmentation of RTSs along the QTEC using Sentinel-2 data.

## 2. Related Work

Previous studies have adopted deep learning methods to extract RTSs from remote sensing imagery and have verified the effectiveness and feasibility of automated RTS mapping [47,48,49,50]. These studies include CubeSat-based RTS mapping, transfer learning-based RTS mapping, and deep neural network-based RTS extraction in different permafrost regions. More recently, Rodenhizer et al. compared different satellite imagery sources for automated RTS detection, highlighting the influence of image resolution and sensor selection on RTS mapping performance [56]. Wu et al. proposed a hybrid CNN–Transformer network for RTS recognition in the central Qinghai–Tibet Plateau, indicating that Transformer-based or hybrid architectures are increasingly being explored for RTS mapping [57].

These existing models can generally be understood from two mainstream architectural perspectives: CNN-based designs and Transformer-based designs. Based on the inherent attributes of these two architectural frameworks, this section discusses their respective potentials and limitations for RTS extraction tasks, especially under the medium-resolution Sentinel-2 imagery setting.

### 2.1. CNN-Based Semantic Segmentation Methods

Fully Convolutional Networks (FCNs) achieved a breakthrough in end-to-end pixel-level prediction by replacing fully connected layers with convolutional layers [58], pioneering the application of deep learning in semantic segmentation. During feature extraction, FCN enhances the ability to expand the receptive field and capture high-level semantic information by virtue of consecutive downsampling. It inevitably reduces feature map resolution, resulting in weakening preservation for localization accuracy and boundary detail.

The U-Net introduces skip connections to combine low-level detail features with high-level semantic features [59]. The DeepLab series adopts dilated convolutions and atrous spatial pyramid pooling (ASPP) to achieve adaptive expansion of the receptive field [60]. High-Resolution Network (HRNet) achieves preservation capability of detail information by extracting features through parallel multi-resolution processes and exchanging information between different resolutions [61].

However, the CNN-based models exhibit inherent limitations in dynamic adaptation capability to regionally variable patterns of segmentation objects due to their hierarchical feature extraction and localized convolution operations. Furthermore, CNN-based models typically employ addition or concatenation operations for feature fusion, lacking a dynamic evaluation mechanism for the importance of features from different levels. These characteristics may limit the adaptability of CNN-based models to RTS segmentation in medium-resolution Sentinel-2 imagery.

### 2.2. Transformer-Based Semantic Segmentation Methods

Vision Transformer (ViT) has pioneered a new paradigm for visual tasks by dividing images into patch sequences and applying self-attention mechanisms [62]. ViT exhibits capability in evaluating feature relationships across the global scope at the expense of a high computational complexity of O(n^2^) relative to sequence length n. Shifted Window (Swin) Transformer achieves linear O(n) complexity through local window self-attention and shifting operations [63]. Additionally, its hierarchical architecture and better inductive biases make it particularly suitable for dense prediction tasks, though the windowed mechanism may limit direct global feature interactions.

Transformer-based architectures have also been increasingly adopted in remote sensing semantic segmentation, such as Efficient Transformer and UNetFormer [64,65]. These studies indicate that Transformer-based designs are effective for modeling contextual information. However, directly applying a general Transformer-based segmentation model may still be insufficient for RTS delineation in medium-resolution Sentinel-2 imagery, because RTSs often show complex morphology, irregular boundaries, and weak contrast with surrounding backgrounds. Therefore, this study adopts the Swin Transformer as the backbone, but further designs task-specific feature enhancement and decoder modules to improve target–background discrimination, contextual refinement, and boundary recovery for RTS identification.

## 3. Data and Methods

### 3.1. Study Region

**Figure 1:**
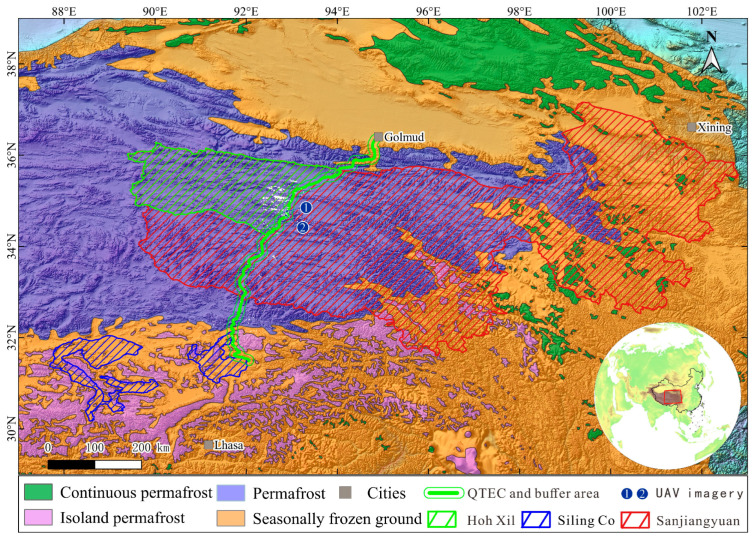
Location of study region. White area near to QTEC is location of RTS dataset [66].

The QTEC has an extremely significant role in economic development, social stability and culture exchanges for three major linear infrastructures (i.e., Qinghai–Tibet Railway, Qinghai–Tibet Highway, and Qinghai–Tibet DC power transmission line). The QTEC crosses the Hoh Xil National Nature Reserve, the Sanjiangyuan National Nature Reserve, and the Siling Co National Nature Reserve (Figure 1). RTSs developed near QTEC pose significant risks to the critical infrastructure and impact fragile ecosystems. Here, we utilize an existing inventory map of RTSs along the QTEC as the data resource for developing MOCA-Net for RTS identification in this study [66].

### 3.2. Data

The dataset, comprising Sentinel-2 imagery and corresponding RTS labels, covers the period from July to August 2019 [66]. The Sentinel-2 imagery was preprocessed through standard operations including cloud removal, noise reduction, and atmospheric correction. Although NIR/SWIR bands and DEM data are beneficial for RTS mapping, this study used only Sentinel-2 RGB bands as simple and consistent inputs to specifically evaluate the proposed method for RTSs delineation. Integrating additional data would require extra preprocessing steps such as resampling and co-registration, thereby introducing further uncertainties and complexity beyond the scope of this study.

RTS labels from Xia et al. [66] were adopted for dataset construction and displayed as white-filled polygons in the study area map (Figure 1). To generate positive samples, each RTS polygon was first expanded using a buffer distance equal to 10% of its perimeter, and the bounding box of the buffered polygon was then used to crop the corresponding Sentinel-2 image patch. This ensured complete RTS coverage and sufficient surrounding contextual background. The corresponding binary masks were generated from the original RTS polygons. Negative samples were selected from areas without mapped RTSs to construct a balanced dataset. The patch size of each sample varies with the scale of each RTS. The final dataset contains 1748 samples, including 874 positive samples and 874 negative samples.

Because many RTSs are small, they usually occupy only a limited number of pixels in the original 10 m Sentinel-2 imagery. As a result, their shapes and boundaries are often not clear. To make the RTS regions in the model input easier to identify, we used a pre-trained ESRGAN model to enlarge the cropped Sentinel-2 images by 4 times [67], from 10 m to 2.5 m. It should be noted that this step was used only as an image-enhancement process before model input, and it does not mean that real 2.5 m satellite observations were generated. Because the original 10 m imagery does not contain real ground details at this finer scale, ESRGAN may add some details that do not exist in the original image, thereby producing artifacts. Therefore, the influence of ESRGAN on the segmentation results should be interpreted with caution. For spatial consistency, the corresponding segmentation masks were upsampled using nearest-neighbor interpolation to match the enhanced remote sensing imagery. In addition, because the masks were upsampled using nearest-neighbor interpolation, staircase-like boundary effects may appear in the enlarged labels. Before model training, all image patches and masks were resized to 224 × 224 pixels. All compared models were trained and evaluated under the same ESRGAN-enhanced preprocessing pipeline and the same upsampled labels to ensure comparability across models.

### 3.3. Methodology for MOCA-Net Design

**Figure 2:**
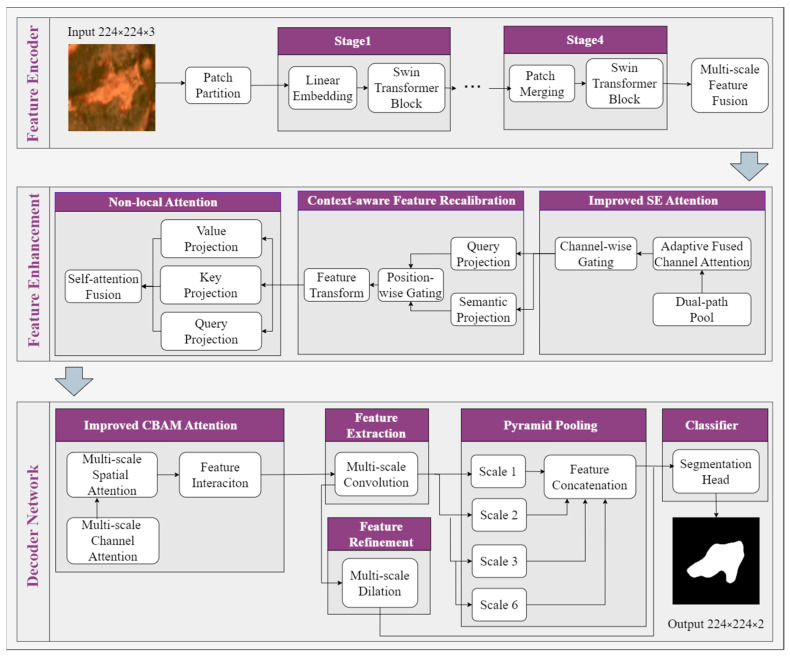
Overall architecture of MOCA-Net, consisting of a Swin Transformer encoder, a Feature Enhancement Network, and an Enhanced Decoder.

**Figure 3:**
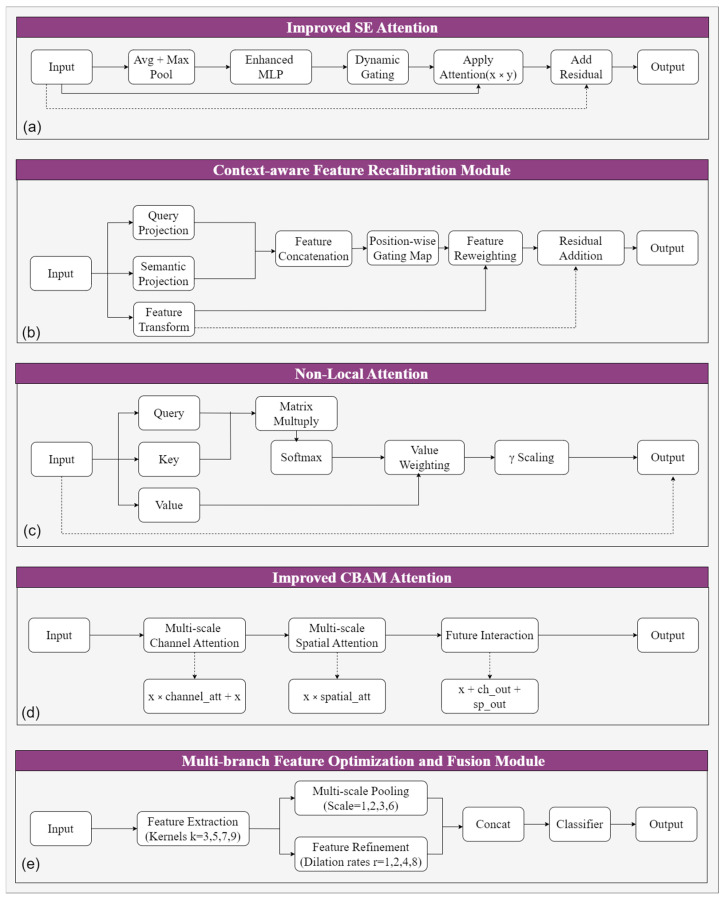
Key modules introduced in MOCA-Net: (**a**) improved SE module; (**b**) Context-aware Feature Recalibration Module; (**c**) Non-local Attention Module; (**d**) improved CBAM module; and (**e**) Multi-branch Feature Optimization and Fusion Module.

The MOCA-Net is built upon the Swin Transformer backbone. As shown in Figure 2, MOCA-Net consists of a feature encoder network, a Feature Enhancement Network and a decoder network. The latter two components of MOCA-Net incorporate several key designs specifically tailored to overcome the aforementioned challenges in RTSs’ identification (Figure 3).

#### 3.3.1. Swin Transformer Encoder

The feature encoder network adopts Swin Transformer as backbone. It extracts hierarchical multiple-scale features through four progressive stages containing 2, 2, 18 and 2 Swin Transformer blocks, respectively. Each block employs window-based self-attention and shifted window operation to capture both local details and global dependencies. In Stage 1, the input image is partitioned into 4 × 4 patches and converted into initial feature representations via linear embedding. Stages 2–4 progressively downsample the feature maps via patch merging, while simultaneously expanding the channel dimension at each level. Notably, Stage 3 contains 18 Transformer blocks, enabling the extraction of richer high-level semantic features. Finally, multi-scale features with rich semantic information from four stages are fused for subsequent feature enhancement (Figure 2).

#### 3.3.2. Feature Enhancement Network

Although the Swin encoder is capable of encoding rich contextual information through its window-based self-attention mechanism, its receptive scope remains constrained by fixed local windows. Considering the variable sizes of RTSs, it remains necessary to capture long-range dependencies across spatially distant regions. To address this, a Feature Enhancement Network is constructed by integrating the improved SE module, the Context-aware Feature Recalibration Module, and the Non-local Attention Module. The improved SE module is used to enhance target-related feature representations via channel-wise recalibration. The Context-aware Feature Recalibration Module applies gated recalibration and discriminative refinement to the already-encoded contextual features. The Non-local Attention Module explicitly strengthens the modeling of long-range dependency by global feature interactions. Then, the enhanced features are passed to the decoder network for subsequent boundary recovery and pixel-wise classification.

Improved SE Attention Module

**Figure 4:**
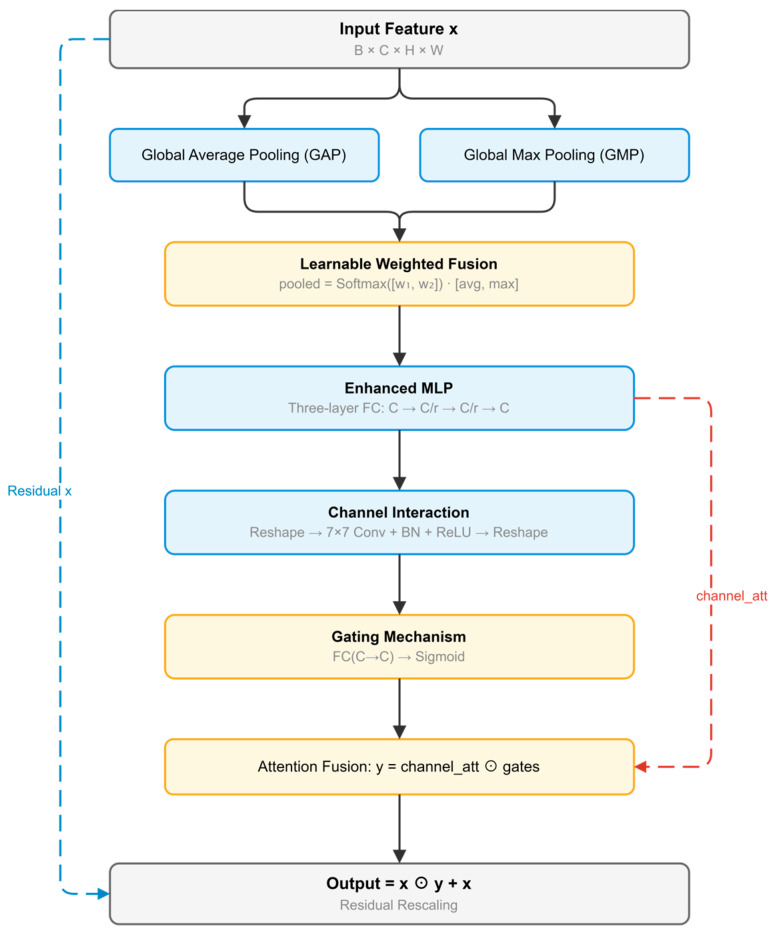
Improved SE Attention Module.

Building upon the foundational concepts from SENet [68] while incorporating efficient channel interaction strategies inspired by ECA-Net [69], this paper improves the traditional Squeeze-and-Excitation (SE) module through several key modifications (Figure 4). The improved SE module introduces dual-path pooling that combines global average pooling and max pooling to capture different statistical characteristics of channel features. An enhanced Multi-Layer Perceptron (MLP) structure is adopted for non-linear feature transformation. Additionally, dynamic fusion mechanisms with learnable weights and channel interaction modeling inspired by ECA-Net’s efficient channel attention are incorporated to achieve more refined feature selection and improve the model’s representational capacity.

(1) $$z_{avg}=GAP(X),z_{max}=GMP(X)$$

(2) $$\alpha_{avg},\alpha_{max}=Softmax(\omega_{avg},\omega_{max})$$

(3) $$z=\alpha_{avg}z_{avg}+\alpha_{max}z_{max}$$

(4) s=MLP(z)

(5) $$g=\sigma (W_{g}\psi (s)+b_{g})$$

(6) $$X_{SE}=X⊙reshape(s⊙g)+X$$

For an input feature map X, z_avg_ and z_max_ denote the global average-pooled and max-pooled channel descriptors, respectively. α_avg_ and α_max_ are learnable fusion weights, ψ(·) denotes the channel interaction operation, and ⊙ denotes element-wise multiplication. In this way, the improved SE module performs dual-path pooling, dynamic fusion, channel interaction, and residual channel recalibration.

The pseudocode of the improved SE Attention Module is summarized in Algorithm 1.

**Algorithm** **1.** Pseudocode of the Improved SE Attention ModuleInput: feature map X ∈ ℝ^{B×C×H×W}^Output: recalibrated feature map X_SE ∈ ℝ^{B×C×H×W}^1. Compute z_avg = GAP(X) and z_max = GMP(X).2. Obtain learnable fusion weights α_avg and α_max by softmax normalization.3. Fuse the two channel descriptors to form z = α_avg z_avg + α_max z_max.4. Feed z into the enhanced MLP to obtain channel attention features s.5. Apply channel interaction modeling ψ(·) to s.6. Generate the gating vector g from the interacted features through a linear transform and sigmoid mapping.7. Combine s and g by element-wise multiplication to form the final channel recalibration weights.8. Reweight X channel-wise and add the residual connection to obtain X_SE.

Context-aware Feature Recalibration Module

By virtue of the Swin Transformer encoder’s hierarchical architecture and window-based self-attention mechanism, the input features to the Context-aware Feature Recalibration Module (CFRM) already contain rich spatial contextual information. Accordingly, rather than introducing additional region-level context aggregation, the proposed CFRM is designed to modulate and recalibrate the already-encoded contextual features. Thus, the CFRM can enhance feature discriminability and suppress interference from similar background regions.

**Figure 5:**
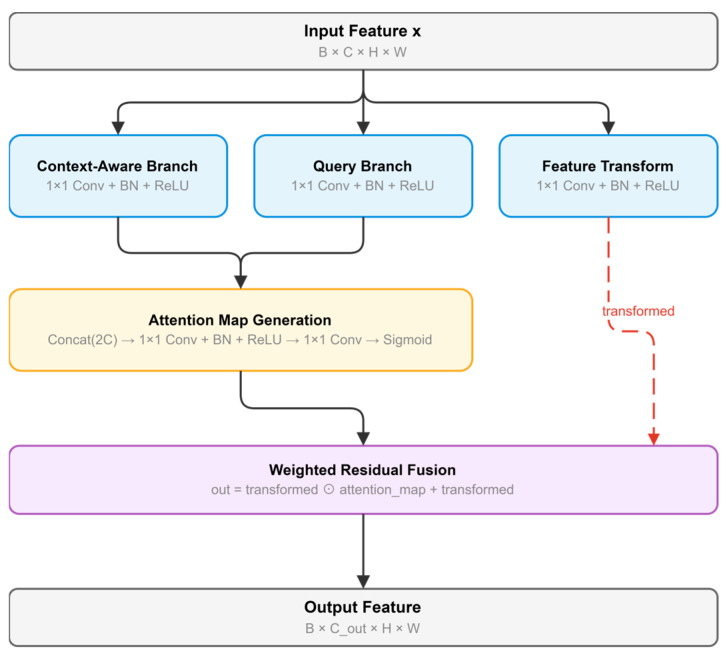
Context-aware Feature Recalibration Module.

The CFRM takes the input feature and processes it through three parallel branches (Figure 5). Two of the branches generate complementary semantic representations from different transformation perspectives, while the third one produces the base features required for subsequent fusion. The first two outputs are concatenated along the channel dimension. A lightweight convolutional layer followed by a Sigmoid activation is then applied to generate a spatial weight map, which evaluates the importance of each spatial location under the current contextual representation. This weight map is subsequently multiplied element-wise with the base features from the third branch. The recalibrated features are then combined with the transformed base representation through a residual-like pathway, yielding the final output of the module.

It is worth noting that the CFRM focuses on recalibrating already-encoded contextual features, rather than explicitly performing global context aggregation. The complementary modeling of global long-range dependencies is handled by the subsequent Non-local Attention Module.

(7) $$F_{c}=\phi_{c}(X),F_{q}=\phi_{q}(X),F_{t}=\phi_{t}(X)$$

(8) $$A=\sigma (\psi ([F_{c};F_{q}]))$$

(9) $$X_{CFRM}=F_{t}⊙A+F_{t}$$

For an input feature map X, F_c_, F_q_, and F_t_ denote the outputs of three parallel feature transformation branches, respectively. [;] denotes channel-wise concatenation, ψ(·) denotes the lightweight convolution used to generate the spatial recalibration map, A is the resulting spatial weight map, and ⊙ denotes element-wise multiplication. In this way, the CFRM adaptively modulates the already-encoded contextual features while preserving the transformed base representation through a residual pathway.

The pseudocode of the Context-aware Feature Recalibration Module is summarized in Algorithm 2.

**Algorithm** **2.** Pseudocode of the Context-aware Feature Recalibration ModuleInput: feature map X ∈ ℝ^{B×C×H×W}^Output: recalibrated feature map X_CFRM ∈ ℝ^{B×C×H×W}^1. Feed X into the context-aware branch to obtain F_c.2. Feed X into the query branch to obtain F_q.3. Feed X into the transform branch to obtain F_t.4. Concatenate F_c and F_q along the channel dimension.5. Generate the spatial recalibration map A by applying a lightweight convolution and sigmoid activation to the concatenated features.6. Recalibrate F_t by element-wise multiplication with A.7. Add F_t through a residual-like pathway to obtain X_CFRM.8. Return X_CFRM.

Non-local Attention Module

While the Swin Transformer encoder provides efficient hierarchical attention through windowed self-attention and shifted window mechanisms, achieving global coverage through multi-layer indirect information propagation, this module introduces direct global attention to ensure comprehensive long-range dependency modeling without multi-hop limitations. Although this introduces quadratic complexity, direct global modeling is adopted to ensure comprehensive feature interaction.

**Figure 6:**
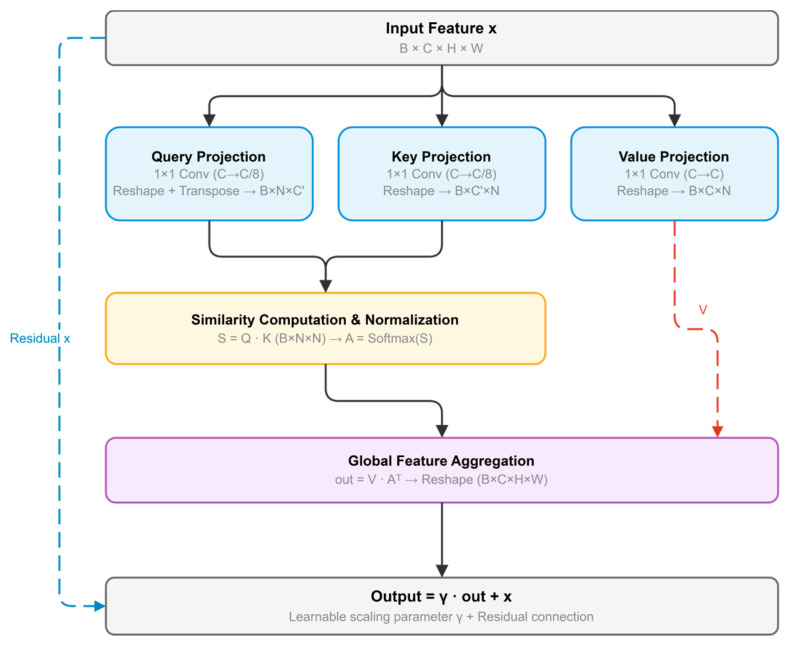
Non-local Attention Module.

Following the self-attention framework from non-local attention [70], this paper introduces a Non-local Attention Module that enhances the model’s ability to model correlations between distant regions through global feature interaction (Figure 6). The module generates Query, Key, and Value features through 1 × 1 convolutions, with Query and Key compressed to reduce computational complexity.

Global feature aggregation is performed through attention weight computation and matrix multiplication, creating contextual representations that incorporate weighted information from across the entire feature map. The module includes learnable scaling parameters and residual connections to maintain feature integrity while enhancing long-range dependency modeling for RTS segmentation.

(10) $$Q=W_{q}\times X,K=W_{k}\times X,V=W_{v}\times X$$

(11) $$a_{ij}=\frac{exp(Q_{i}^{T}K_{j})}{\sum_{j}exp(Q_{i}^{T}K_{j})}$$

(12) $$Y_{i}=\gamma \sum_{j}a_{ij}V_{j}+X_{i}$$

For an input feature map X, Q, K, and V denote the Query, Key, and Value feature representations generated by three 1 × 1 convolutions, respectively. a_ij_ denotes the normalized affinity between spatial positions i and j, and γ is a learnable scaling parameter. Through this formulation, the Non-local Attention Module performs direct global feature interaction and enhances long-range dependency modeling beyond the local attention scope of the Swin encoder.

The pseudocode of the Non-local Attention Module is summarized in Algorithm 3.

**Algorithm** **3.** Pseudocode of the Non-local Attention ModuleInput: feature map X ∈ ℝ^{B×C×H×W}^Output: enhanced feature map Y ∈ ℝ^{B×C×H×W}^1. Generate the Query feature Q from X using a 1 × 1 convolution.2. Generate the Key feature K from X using a 1 × 1 convolution.3. Generate the Value feature V from X using a 1 × 1 convolution.4. Reshape Q, K, and V for global attention computation.5. Compute the pairwise affinity matrix between spatial positions using Q and K.6. Normalize the affinity matrix with softmax to obtain the attention weights A.7. Aggregate global contextual information by weighted multiplication between V and A.8. Reshape the aggregated feature to the original spatial layout.9. Fuse the aggregated feature with X through a learnable scaling parameter γ and a residual connection to obtain Y.

#### 3.3.3. Enhanced Decoder

This paper designs a cascaded multi-component decoder structure. This decoder consists of two key modules: an improved CBAM Attention Module for feature importance modeling, and a Multi-branch Feature Optimization and Fusion Module for multi-scale processing and refinement.

Improved CBAM Attention Module

**Figure 7:**
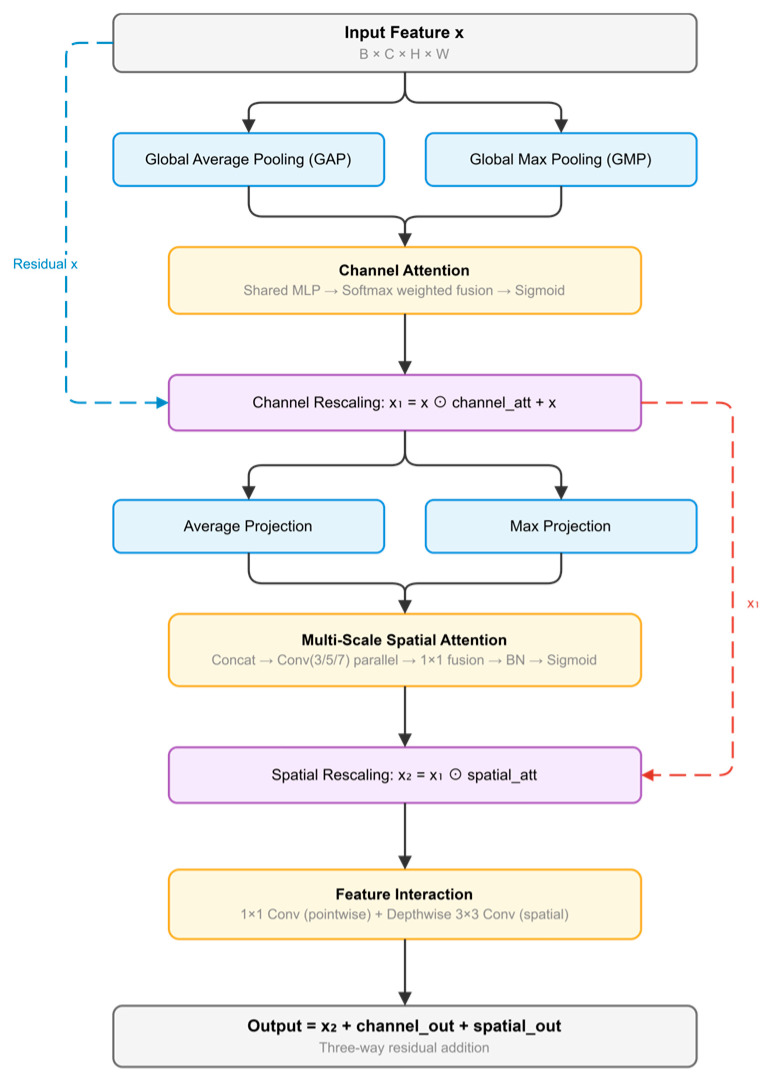
Improved CBAM Attention Module.

Building upon CBAM [71] with multi-scale fusion approaches motivated by AFF [72], this paper improves the traditional Convolutional Block Attention Module (CBAM) through enhanced multi-scale processing capabilities (Figure 7). The improved module incorporates multi-scale channel attention that combines adaptive pooling operations with learnable weight parameters for dynamic channel importance modeling. Multi-scale spatial attention is enhanced through multi-receptive field convolutions to better capture spatial dependencies across different scales.

Additionally, a feature interaction component adopts dual-branch processing to model inter-channel relationships and spatial context through parallel pathways. These enhancements aim to address the complex morphology and multi-scale characteristics typical of RTS regions.

(13) $$M_{c}(X)=\sigma (\lambda_{1}MLP(GAP(X))+\lambda_{2}MLP(GMP(X)))$$

(14) $$X_{c}=X⊙M_{c}(X)+X$$

(15) $$S(X_{c})=[{Avg}_{c}(X_{c});{Max}_{c}(X_{c})]$$

(16) $$M_{s}(X_{c})=\sigma (f^{1\times 1}([f^{3\times 3}(S(X_{c}));f^{5\times 5}(S(X_{c}));f^{7\times 7}(S(X_{c}))]))$$

(17) $$X_{s}=X_{c}⊙M_{s}(X_{c})$$

(18) $$X_{CBAM}=\Phi_{int}(X_{s})$$

(19) $$\Phi_{int}(X)=X+\phi_{ch}(X)+\phi_{sp}(X)$$

For an input feature map X, M_c_(X) and M_s_(X_c_) denote the channel attention map and spatial attention map, respectively. λ_1_ and λ_2_ are learnable fusion weights, [;] denotes channel-wise concatenation, and ⊙ denotes element-wise multiplication. Φ_int_(·) denotes the feature interaction module, where ϕ_ch_(·) and ϕ_sp_(·) represent the channel-mixing branch and the spatial refinement branch, respectively. In this way, the improved CBAM performs dynamic channel recalibration, multi-scale spatial attention modeling, and subsequent feature interaction refinement to enhance feature discrimination for RTS segmentation.

The pseudocode of the improved CBAM Attention Module is summarized in Algorithm 4.

**Algorithm** **4.** Pseudocode of the Improved CBAM Attention ModuleInput: feature map X ∈ ℝ^{B×C×H×W}^Output: refined feature map X_CBAM ∈ ℝ^{B×C×H×W}^1. Compute global average-pooled and max-pooled channel descriptors from X.2. Generate the channel attention map M_c(X) by learnable weighted fusion and sigmoid activation.3. Recalibrate X channel-wise to obtain X_c.4. Compute the average and max spatial projections from X_c and concatenate them.5. Apply parallel multi-scale convolutions to the concatenated spatial descriptor.6. Fuse the multi-scale spatial responses and generate the spatial attention map M_s(X_c) by sigmoid activation.7. Reweight X_c spatially to obtain X_s.8. Feed X_s into the feature interaction module.9. Compute the channel-mixing branch and spatial refinement branch in parallel, and fuse them with X_s by residual addition to obtain X_CBAM.10. Return X_CBAM.

Multi-branch Feature Optimization and Fusion Module

**Figure 8:**
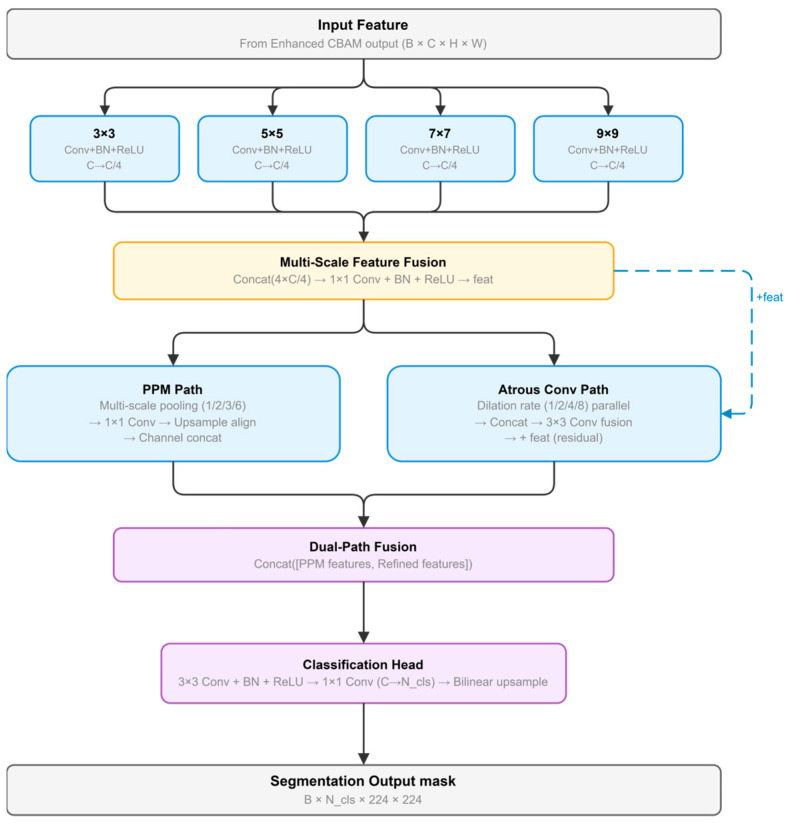
Multi-branch Feature Optimization and Fusion Module.

To enhance feature expression capability at the decoder stage, this paper designs a multi-branch architecture with parallel processing pathways (Figure 8). The module employs multi-scale convolution branches to capture local details and contextual information at different scales, with results concatenated for enhanced base feature generation.

Building on this foundation, the Pyramid Pooling Module (PPM) and Refinement Module operate in parallel to provide complementary information. The PPM captures multi-scale global context through adaptive pooling operations, while the Refinement Module employs dilated convolutions to expand receptive fields and enhance spatial dependency modeling. The multi-scale contextual features and enhanced spatial features are then concatenated to guide final pixel classification, combining global context aggregation with local detail preservation for RTS segmentation.

(20) $$F_{b}=\phi ([f_{3}{(X}_{CBAM});f_{5}(X_{CBAM});f_{7}(X_{CBAM});f_{9}(X_{CBAM})])$$

(21) $$F_{PPM}=[Up(P_{1}(F_{b}));Up(P_{2}(F_{b}));Up(P_{3}(F_{b}));Up(P_{6}(F_{b}))]$$

(22) $$F_{Ref}={R(F}_{b})+F_{b}$$

(23) $$Y=Up(Conv({[F}_{PPM};F_{Ref}]))$$

For the decoder input feature X_CBAM_, f_k_(·) denotes convolution with kernel size k, φ(·) denotes the fusion operation for multi-scale local features, P_s_(·) denotes pyramid pooling at scale s, R(·) denotes the refinement branch with atrous convolutions, and Up(·) denotes bilinear upsampling. Through this formulation, the decoder combines multi-scale local feature extraction, global context aggregation, and detail refinement to generate the final segmentation output Y.

The pseudocode of the Multi-branch Feature Optimization and Fusion Module is summarized in Algorithm 5.

**Algorithm** **5.** Pseudocode of the Multi-branch Feature Optimization and Fusion ModuleInput: decoder feature X_CBAM ∈ ℝ^{B×C×H×W}^Output: segmentation output Y1. Feed X_CBAM into parallel multi-scale convolution branches with different kernel sizes.2. Concatenate the branch outputs and fuse them to obtain the base feature F_b.3. Feed F_b into the Pyramid Pooling Module at multiple scales.4. Upsample all pooled features to the same spatial resolution and concatenate them to form F_PPM.5. Feed F_b into the refinement branch with atrous convolutions to obtain refined spatial features.6. Add the residual connection from F_b to the refinement output to form F_Ref.7. Concatenate F_PPM and F_Ref to combine global contextual and refined spatial information.8. Apply convolution-based classification on the fused feature.9. Upsample the prediction to obtain the final segmentation output Y.

#### 3.3.4. MOCA-Net Design Strategy and Architectural Logic

MOCA-Net adopts a three-tier architecture specifically targeting the challenges in RTS segmentation, including complex morphology, variable sizes, and low contrast and fuzzy boundaries against the surrounding landscape. The design philosophy follows an encoder-enhancer-decoder pipeline to progressively refine feature representations for precise segmentation, with particular emphasis on target–background discrimination, contextual refinement, and boundary recovery.

The Swin Transformer encoder extracts initial feature maps with rich semantic information and multi-scale characteristics through its hierarchical structure and windowed self-attention mechanism. A Feature Enhancement Network is introduced between the encoder and decoder to improve feature discrimination before spatial recovery. The module sequence follows a specific logic: the improved SE module calibrates channel importance; leveraging the context-rich features from the encoder, the Context-aware Feature Recalibration Module recalibrates the already-encoded contextual features to improve feature discrimination and reduces confusion from similar background regions; and complementing the encoder’s windowed attention, non-local attention provides direct global feature interaction to ensure comprehensive dependency modeling without multi-hop limitations.

The Enhanced Decoder focuses on accurate spatial resolution recovery and fine boundary reconstruction. It applies improved CBAM for attention recalibration, extracts multi-scale local features through parallel convolution branches, and employs two complementary parallel strategies: PPM for global context aggregation and the Refinement Module with atrous convolutions for boundary detail delineation. The complementary information from both branches is concatenated to guide final pixel classification.

This structured architecture design, including the Feature Enhancement Network’s internal ordering and the decoder’s parallel multi-scale strategies, targets the concrete challenges in RTS segmentation through coordinated attention mechanisms and feature fusion approaches, achieving comprehensive feature enhancement through strategic module positioning and functional complementarity.

### 3.4. Loss Function and Experimental Settings

RTS Segmentation is a binary semantic segmentation task; thus, the Cross-Entropy Loss is chosen as the optimization objective (24).

(24) $$L_{\mathrm{CE}}=-\frac{1}{N}\sum_{i=1}^{N}\sum_{c=1}^{C}y_{\mathrm{ic}}\log\left(p_{\mathrm{ic}}\right)$$

where N represents the total number of pixels, C represents the number of categories, and y_ic_ indicates the true label of the i-th pixel: y_ic_ = 1 when pixel i belongs to category c; otherwise, y_ic_ = 0. pic represents the model’s predicted probability that the i-th pixel belongs to category c.

The dataset was divided into training, validation, and test sets at a ratio of 70%, 20%, and 10%, respectively. For the main model comparison, the split was generated at the patch level using a fixed random seed of 42. The random seed also affected model initialization, data shuffling, and stochastic data augmentation during training. It should be noted that the split was performed at the patch level rather than at the scene or geographic-block level; therefore, spatial dependence among nearby patches may not be fully eliminated. Because nearby patches may share similar landscape backgrounds, surface textures, and environmental conditions, this patch-level split may lead to somewhat optimistic performance estimates compared with a geographically independent scene- or block-level split. Nevertheless, all compared models were trained and evaluated under the same data split, preprocessing pipeline, and training protocol, so the comparison among different models remains fair under the current experimental setting. Therefore, the reported results should be interpreted as performance under the current patch-level setting rather than as a strict assessment of spatial transferability to fully independent regions. To further assess the robustness of the reported results to random seed variation, additional repeated runs using seeds 62, 82, 102, and 122 were conducted for MOCA-Net and the Swin + Basic Decoder baseline, as reported in Section 4.4.

During the training process, the Adam optimizer is used to minimize loss, with an initial learning rate of 1 × 10^−4^ and a weight decay of 1 × 10^−4^ to prevent overfitting. To avoid gradient explosion, a maximum norm clipping strategy is adopted with a clipping value of 1.0. For learning rate adjustment, the ReduceLROnPlateau scheduler is introduced, which reduces the learning rate by a factor of 0.5 when the validation loss shows no improvement for 5 consecutive epochs. To further avoid overfitting, an early stopping mechanism based on validation loss is employed: if the validation loss does not improve for 10 consecutive epochs, training is terminated early.

The batch size during training is set to 8, with a maximum of 50 training epochs. During training, data augmentation includes random horizontal flipping, random vertical flipping, and random rotation. For transfer learning, the Swin Transformer encoder is initialized with pre-trained weights, while the newly added modules are trained from scratch. Throughout the training process, multiple performance metrics of the model on the validation set are continuously monitored and recorded. The optimal model weights with the lowest validation loss are applied to the test set. All compared models are trained and evaluated under the same data split, preprocessing pipeline, and training protocol to ensure a fair comparison. All model training and accuracy evaluation experiments were conducted on the Kaggle platform in a GPU environment equipped with NVIDIA Tesla T4 hardware.

Although a balanced positive/negative patch dataset was constructed to partially reduce sample-level imbalance, foreground–background imbalance remains inherent at the pixel level because RTS regions usually occupy a relatively small proportion of each patch. In the current study, no explicit pixel-level loss reweighting strategy, such as class-weighted cross-entropy, Dice loss, or focal loss, was introduced to further mitigate this imbalance. Therefore, pixel-level class imbalance remains a limitation of the current study, and future work should evaluate whether loss reweighting or imbalance-aware loss functions can further improve RTS segmentation performance.

### 3.5. Evaluation Metrics

Two types of metrics are selected to evaluate model performance for RTS identification: classification quality metrics and segmentation quality metrics.

Classification quality metrics include precision and recall. Precision measures the proportion of correctly predicted RTS pixels among all pixels predicted as RTS, reflecting the reliability of the model’s predictions. Recall measures the proportion of actual RTS pixels that are correctly detected, reflecting the completeness of the model’s detection. The formulas are as follows.

(25) $$\mathrm{Precision}=\frac{\mathrm{TP}}{\mathrm{TP}+\mathrm{FP}}$$

(26) $$\mathrm{Recall}=\frac{\mathrm{TP}}{\mathrm{TP}+\mathrm{FN}}$$

where TP (True Positive) represents the number of pixels correctly predicted as RTS, FP (False Positive) represents the number of pixels incorrectly predicted as RTS, and FN (False Negative) represents the number of pixels in actual RTS areas that are not detected.

Segmentation quality metrics include Dice coefficient, Intersection over Union (IoU), and Mean Intersection over Union (Mean IoU). The Dice coefficient calculates the similarity between the prediction result and the ground truth. In this binary segmentation task, IoU refers specifically to the RTS-class IoU, whereas Mean IoU denotes the average IoU over the background and RTS classes. For metric computation, the predicted class label of each pixel is determined by selecting the class with the maximum predicted probability, without applying an additional probability threshold. During evaluation, predictions and labels within each mini-batch are flattened to compute batch-level metrics, and the final reported values are obtained by averaging these batch-wise results over the evaluation set. This batch-level averaging strategy was applied consistently to all compared models under the same test split and batch size, so the comparison among models remains fair under the current evaluation protocol. However, compared with metrics computed from a pooled confusion matrix over the entire test set or averaged at the image level, batch-level averaging may introduce slight dependence on batch composition. Therefore, this evaluation strategy is acknowledged as a limitation of the current study, and future work will adopt whole-test-set aggregation or per-image averaging to further improve the robustness and comparability of evaluation results. The formulas are as follows.

(27) $$Dice=\frac{2|X\cap Y|}{\left|X|+|Y\right|}=\frac{2TP}{2TP+FP+FN}$$

(28) $$IoU=|\frac{X\cap Y}{X\cup Y}|=\frac{TP}{TP+FP+FN}$$

(29) $$mIoU=\frac{1}{C}\sum_{i=1}^{c}\frac{TP_{i}}{{TP}_{i}+{FP}_{i}+{FN}_{i}}$$

where X and Y represent the prediction result and the ground truth, respectively. C is the number of categories, and TP_i_, FP_i_, and FN_i_ represent the numbers of true positives, false positives, and false negatives for the i-th category, respectively.

## 4. Results

### 4.1. Model Comparison During Training Process

**Figure 9:**
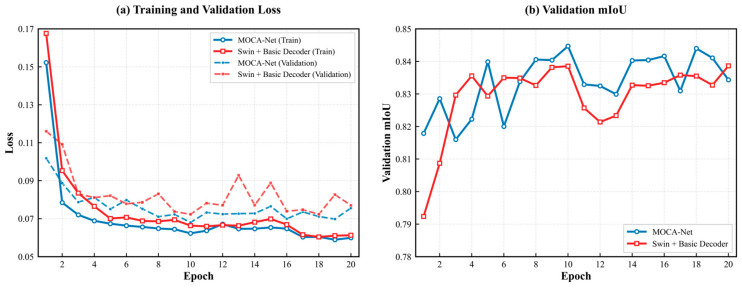
Comparison between MOCA-Net and Swin + Basic Decoder during training. (**a**) Training and validation loss versus epoch. (**b**) Validation mIoU versus epoch.

As shown in Figure 9a, the training and validation loss of MOCA-Net exhibit a smoother convergence trend with smaller gaps between them, indicating that training process is stable and has no obvious overfitting. In comparison, the Swin + Basic Decoder presents greater fluctuations, as evidenced by the validation loss curve in Figure 9a during epochs 8–16. This implies the instability in the training process of Swin + Basic Decoder. In Figure 9b, the mIoU curves also show the improved performance of MOCA-Net compared to Swin + Basic Decoder. The mIoU curve of MOCA-Net stabilizes above 0.83 since epoch 7, with peak performance surpassing 0.84. By comparison, the mIoU curve of Swin + Basic Decoder only stabilizes around 0.83 and never surpassed 0.84. In addition, because of the transfer learning strategy, both MOCA-Net and Swin + Basic Decoder present relatively fast convergence speeds.

### 4.2. Model Comparison by Evaluation Metrics

The six metrics listed in Table 1 are used to evaluate the performance of the CNN-based models (DeepLabv3+ Xception71, FCN ResNet152, U-Net, and HRNet) and Transformer-based models (Vision Transformer, SegFormer, Swin + Basic Decoder, and MOCA-Net). These baseline models were selected to provide representative comparisons across both CNN-based and Transformer-based segmentation paradigms. Specifically, FCN, U-Net, DeepLabv3+, and HRNet represent widely used CNN-based architectures with different feature extraction and fusion strategies, whereas Vision Transformer, SegFormer, and Swin + Basic Decoder represent Transformer-based segmentation models. In particular, Swin + Basic Decoder serves as the most direct baseline for evaluating the effectiveness of the proposed feature enhancement and decoder design, while SegFormer is included as a more recent Transformer-based segmentation baseline. Therefore, the purpose of this comparison is to assess the relative effectiveness of MOCA-Net against representative segmentation frameworks.

As shown in Table 1, Transformer-based models surpass CNN-based models in optimization robustness (lower test loss) and segmentation accuracy (higher mIoU, RTS IoU and Dice). Notably, MOCA-Net performs best among all compared models for these four metrics, with the lowest loss (0.0542), highest mIoU (0.8609), highest RTS IoU (0.7473) and highest Dice (0.8547). While MOCA-Net does not attain the highest precision (0.8785 achieved by SegFormer), it retains competitive precision (0.8516) while achieving higher recall (0.8606), showing balanced performance in detection sensitivity for RTSs.

### 4.3. Effect of ESRGAN Preprocessing

To assess whether the ESRGAN-based super-resolution preprocessing materially influences the reported segmentation performance, we further conducted a controlled comparison using the original-resolution Sentinel-2 inputs without ESRGAN enhancement. In this comparison, the same dataset split, random seed, training protocol, and evaluation procedure were retained, and only the ESRGAN preprocessing step was removed. The experiment was performed for both MOCA-Net and the Swin + Basic Decoder baseline in order to examine whether the effect of ESRGAN is consistent across models.

As shown in Table 2, removing ESRGAN led to a modest but consistent decrease in the overall segmentation performance of both models. For MOCA-Net, the mIoU decreased from 0.8609 to 0.8500 and the RTS-class IoU decreased from 0.7473 to 0.7275. For the Swin + Basic Decoder baseline, the mIoU decreased from 0.8542 to 0.8455 and the RTS-class IoU decreased from 0.7359 to 0.7196. Dice and recall also decreased in both models, whereas precision increased in the non-ESRGAN setting. This pattern suggests that removing ESRGAN made the predictions more conservative, thereby reducing commission errors but increasing omission errors, as reflected by the lower recall, Dice, mIoU, and RTS-class IoU.

At the same time, MOCA-Net remained superior to the Swin + Basic Decoder baseline under both the ESRGAN-enhanced and non-ESRGAN settings. This indicates that the performance improvement of MOCA-Net does not come entirely from ESRGAN preprocessing, but is also related to the feature enhancement and decoder design of the model itself. It should be emphasized that ESRGAN only enlarges the original 10 m Sentinel-2 imagery to an input size corresponding to 2.5 m, and it cannot reconstruct genuine 2.5 m surface details from the original 10 m Sentinel-2 imagery. Because such fine-scale ground information is not contained in the original 10 m imagery, ESRGAN may generate some texture or boundary details that look clearer but may not physically exist. Therefore, the performance improvement obtained with ESRGAN should be understood as the effect of an image-enhancement preprocessing step under the current experimental setting, rather than as evidence that Sentinel-2 imagery has obtained a true 2.5 m spatial resolution. Accordingly, the reliance on ESRGAN-enhanced inputs should be regarded as a limitation of the current experimental setting, rather than as a replacement for true high-resolution remote sensing imagery.

### 4.4. Stability Analysis and Statistical Comparison Across Random Seeds

To further evaluate the stability of the reported performance, we repeated the experiments under the ESRGAN-enhanced setting using five random seeds, namely 42, 62, 82, 102, and 122. Considering the computational cost, this repeated-run analysis was conducted for MOCA-Net and the Swin + Basic Decoder baseline. The detailed results of all ten runs are reported in Table 3. Here, the random seed controls both the random train/validation/test split and stochastic factors in training, such as parameter initialization, mini-batch shuffling, and random data augmentation. Therefore, repeating the experiments with different seeds helps assess whether the observed performance gain is robust to both data-partition variation and training randomness rather than arising from a favorable single run.

As shown in Table 3, MOCA-Net consistently outperformed the Swin + Basic Decoder across all five random seeds. Specifically, MOCA-Net achieved higher mIoU, RTS-class IoU, and Dice in every repeated run, indicating that the observed improvement did not arise from a favorable single seed. The corresponding mean and standard deviation values are also listed in Table 3, showing relatively small variations across repeated runs for both models.

To further strengthen the statistical interpretation, Table 4 summarizes the repeated-run results in terms of mean ± standard deviation, 95% confidence intervals of the mean, and paired *t*-test *p*-values for the core segmentation metrics. MOCA-Net achieved a mean mIoU of 0.8595 ± 0.0012, an RTS-class IoU of 0.7471 ± 0.0028, and a Dice coefficient of 0.8546 ± 0.0019, compared with 0.8529 ± 0.0013, 0.7353 ± 0.0029, and 0.8468 ± 0.0021 for the Swin + Basic Decoder, respectively. Paired *t*-tests across the five seeds further indicated that the improvements in mIoU, RTS-class IoU, and Dice were statistically significant (all *p* < 0.001).

Overall, these repeated experiments provide additional evidence that the performance gain of MOCA-Net over the Swin + Basic Decoder baseline is stable and statistically supported under different random settings.

### 4.5. Model Comparison by Visual Inspection of Segmentation Effect

While quantitative metrics have confirmed the effectiveness of the proposed model, visual comparisons with ground truth provide a more intuitive and distinct illustration of its advantages.

#### 4.5.1. Sentinel-2 Imagery

**Figure 10:**
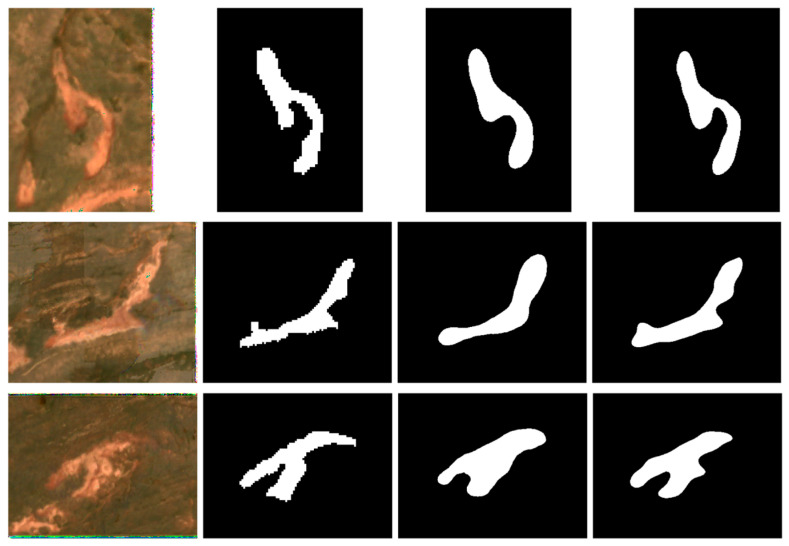
Representative RTS examples with clearer boundaries and stronger target–background contrast. From left to right: ESRGAN-enhanced input, label, Swin + Basic Decoder result, and MOCA-Net result. Visual comparison focuses on boundary delineation and preservation of RTS morphology.

**Figure 11:**
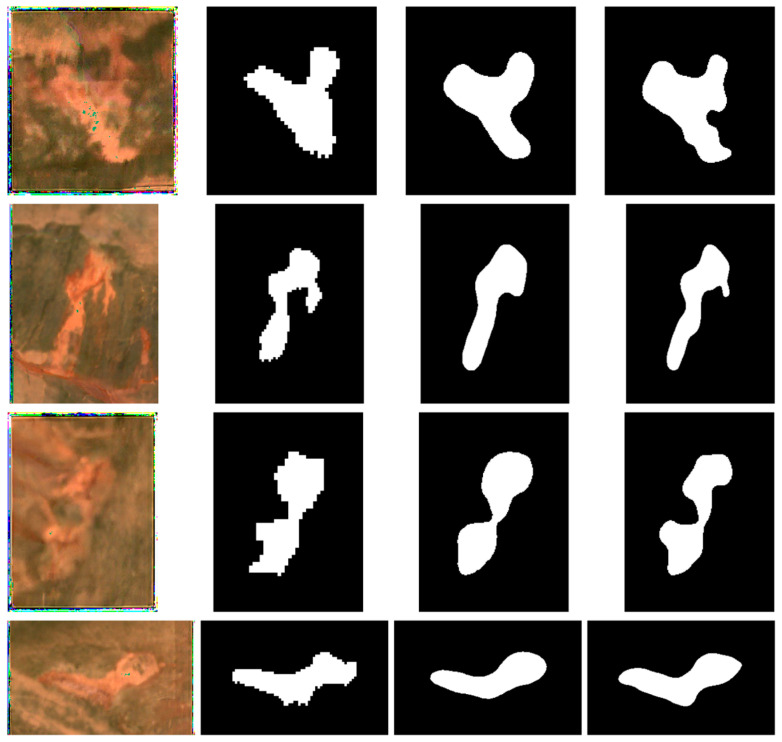
Representative RTS examples with lower target–background contrast and more ambiguous boundaries. From left to right: ESRGAN-enhanced input, label, Swin + Basic Decoder result, and MOCA-Net result. Visual comparison focuses on the handling of challenging boundaries, irregular morphology, and target–background confusion.

To visually compare the segmentation performance of MOCA-Net and Swin + Basic Decoder on Sentinel-2 imagery, seven representative samples are presented in Figure 10 and Figure 11. These examples are intended to illustrate typical visual differences in boundary delineation, preservation of RTS morphology, and target–background confusion. The three samples in Figure 10 show clearer boundaries and more obvious contrast between RTSs and their surrounding landscape, whereas the samples in Figure 11 present more ambiguous transitions and less distinct edges. Although both models capture the general morphology of the seven morphologically diverse samples, MOCA-Net demonstrates superior boundary precision and better preservation of morphological details across all samples. For example, in the first instance shown in Figure 10, a distinct upward-concave morphological feature is visible in both the Sentinel-2 imagery and the corresponding RTS ground truth. Compared with result of Swin + Basic Decoder, the MOCA-Net delineates the curved structure more precisely. In the second example shown in Figure 10, the Swin + Basic Decoder also fails to identify the upward-protruding part of the lower-left RTS area. In the first example of Figure 11, the MOCA-Net also identifies a finer left-concave feature on the lower-right side of the RTS, which is not present in the reference label. This further supports the effectiveness of MOCA-Net in segmentation of RTSs and also implies the effectiveness of the designed Feature Enhancement Network and Enhanced Decoder components. Notably, the more accurate segmentation of RTSs—reflected by finer boundary delineation and more precise morphological representation—is of great significance for the long-term analysis of RTS change. The advantages of MOCA-Net revealed by visual inspection of the segmentation results are more evident than those reflected by the evaluation metrics.

#### 4.5.2. UAV Imagery

**Figure 12:**
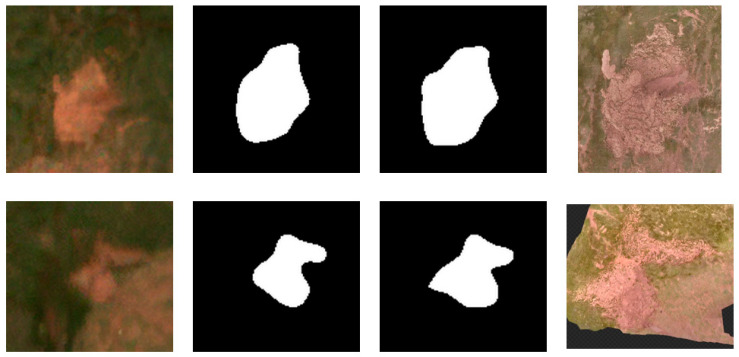
Representative qualitative comparison of segmentation results between MOCA-Net and Swin + Basic Decoder using UAV orthophotos from two sites. From left to right: ESRGAN-enhanced input, Swin + Basic Decoder result, MOCA-Net result, and higher-resolution UAV orthophoto reference.

While remote sensing imagery provides efficient coverage for large-scale RTS mapping, labels derived from image interpretation may contain uncertainties. Therefore, we compared segmentation results between MOCA-Net and Swin + Basic Decoder using UAV orthophotos from two sites adjacent to the Beiluhe section of the QTEC during the summer of 2024 (Figure 1). The centimeter-level UAV imagery serves as a higher-resolution qualitative reference for visual comparison (Figure 12). Site 1 represents a typical elliptical RTS morphology, while Site 2 exhibits irregular complex structures. The UAV orthophotos correspond to the same RTS sites as the Sentinel-2 patches shown in Figure 12 and are used here only as qualitative reference data. Because RTS morphology and boundaries may evolve over time, the Sentinel-2 patches used in Figure 12 were independently extracted from summer 2024 imagery for these two sites, with sufficient surrounding context retained to facilitate visual comparison with the UAV orthophotos.

The comparison suggests that MOCA-Net better delineates RTS boundaries in these two examples. For Site 1, MOCA-Net produces a more geometrically accurate elliptical contour compared to the baseline model. For Site 2, MOCA-Net better captures the overall contour, showing improved capability in handling irregular RTS boundaries across different morphological types.

Although MOCA-Net identifies RTS objects and delineates relatively precise boundaries using Sentinel-2 imagery, discernible discrepancies in fine-scale details remain when compared with the centimeter-level UAV imagery. By comparing the Sentinel-2 and UAV imagery, it is evident that these limitations are primarily attributable to the inherent resolution of the input satellite data rather than algorithmic deficiencies. Therefore, the UAV comparison should be interpreted as a qualitative visual check rather than a formal quantitative validation. Nevertheless, the segmentation results indicate that MOCA-Net can provide useful RTS delineation for large-scale mapping and long-term spatiotemporal analysis.

### 4.6. Ablation Study and Mechanism Analysis

To verify the effectiveness of our proposed components, we conducted ablation experiments comparing four model configurations: (1) Baseline M0 (Swin + Basic Decoder), (2) M1 (adding Feature Enhancement Network), (3) M2 (adding Enhanced Decoder), and (4) M3 (MOCA-Net with both components) (Table 5).

The results show that each component contributes to performance improvements. M1 (Feature Enhancement Network) achieved mIoU of 0.8584 and improved precision to 0.8581, though with slightly lower recall. M2 (Enhanced Decoder) reached mIoU of 0.8572 with the highest precision (0.8628) but more noticeable recall reduction.

The complete model M3 achieved the best overall performance with mIoU of 0.8609 and RTS IoU of 0.7473. Most importantly, M3 maintained a better balance between precision (0.8516) and recall (0.8606), effectively integrating the strengths of both components.

The ablation results demonstrate that both the Feature Enhancement Network and the Enhanced Decoder contribute positively to the model’s overall segmentation performance. While the Feature Enhancement Network yields more balanced improvements across various evaluation metrics, the Enhanced Decoder attains the highest precision at the cost of a more pronounced drop in recall. When integrated, MOCA-Net achieves the best overall performance across all metrics and exhibits a well-balanced precision–recall trade-off. These findings suggest that the two proposed components are complementary and jointly effective for the task of RTS segmentation.

**Figure 13:**
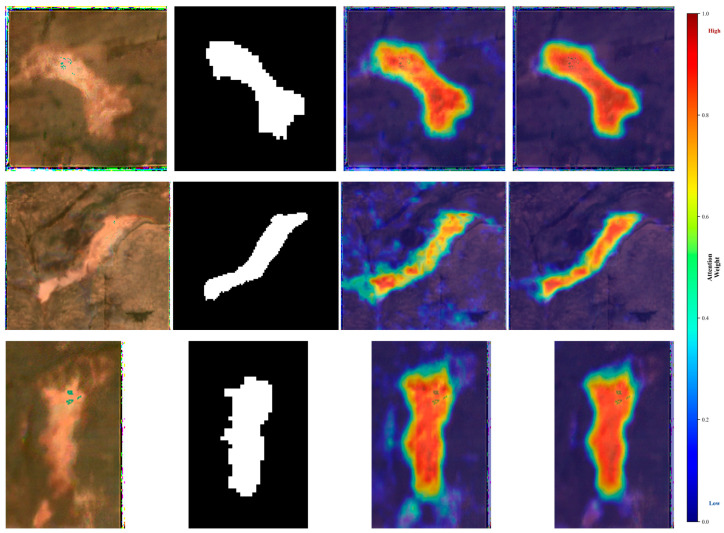
Visualization of learned attention responses for representative RTS samples. From left to right: ESRGAN-enhanced input, label, local Attention (improved SE block), and global attention (Non-local block with CFRM). The colorbar on the right indicates normalized attention weight, ranging from low (blue) to high (red).

To further interpret the performance differences observed in Table 5, particularly the contribution of the Feature Enhancement Network, we visualized the learned attention responses in Figure 13.

The local Attention maps (third column), produced by the improved SE block, mainly highlight visually salient target-related regions and fine-scale feature responses. This behavior is consistent with the channel-wise reweighting mechanism of the SE block, which may help strengthen discriminative spectral–textural cues. However, the local attention still exhibits spurious activations in surrounding background regions (most noticeable in the third row), suggesting that relying solely on local cues may be insufficient to fully suppress context-induced ambiguity in Sentinel-2 imagery.

In contrast, the global attention maps (fourth column), derived from the CFRM and the Non-local block, exhibit more spatially coherent and target-concentrated activation patterns. This observation is qualitatively consistent with the role of global context aggregation and long-range dependency modeling, which may help reduce irrelevant background responses.

Overall, the visual differences between the local and global attention maps qualitatively suggest that combining both mechanisms may contribute to more coherent target-focused feature responses. These visualizations are intended as illustrative qualitative evidence and should not be interpreted as direct proof of causal mechanism.

### 4.7. Computational Complexity Analysis

To further assess whether the performance gain justifies the additional model complexity, we compared MOCA-Net with the Swin + Basic Decoder baseline in terms of both segmentation accuracy and computational cost (Table 6).

As shown in Table 6, MOCA-Net contains 111.744 M trainable parameters, compared with 92.594 M for the Swin + Basic Decoder baseline. Its computational complexity also increases substantially, from 17.793 G to 89.031 G FLOPs. Under the same hardware setting, the average inference time increases from 32.962 ms/image to 38.059 ms/image. Although the FLOPs increase markedly, the increase in measured inference time remains relatively moderate under the tested hardware setting, suggesting that the additional computations do not translate proportionally into runtime overhead in the tested environment. Although the absolute performance improvement over the baseline is moderate, particularly a 1.55% increase in RTS-class IoU, the gain is consistently reflected in both quantitative metrics and qualitative boundary delineation. These results indicate that MOCA-Net achieves improved RTS segmentation at the cost of increased computational complexity, and the practical value of this trade-off should be considered according to the computational resources and deployment requirements of large-scale applications. Therefore, MOCA-Net may be more suitable for applications where boundary accuracy is prioritized, whereas the Swin + Basic Decoder remains a more efficient option for resource-constrained large-scale screening.

## 5. Conclusions

Segmenting RTSs in medium-resolution satellite imagery is challenging due to their complex morphology, variable sizes, and, more importantly, their low contrast and fuzzy boundaries against the surrounding landscape. To address these challenges, this paper proposes MOCA-Net, which employs a three-stage cascaded architecture comprising an encoder, a Feature Enhancement Network, and an Enhanced Decoder to improve target–background discrimination, contextual refinement, and boundary recovery.

Compared to the baseline Swin + Basic Decoder, MOCA-Net achieves improvements in mIoU (from 0.8542 to 0.8609, +0.78%) and IoU (from 0.7359 to 0.7473, +1.55%). The ablation experiments demonstrate the effectiveness of the proposed components. Visual comparisons with both labels and UAV imagery show that MOCA-Net produces more accurate segmentation results for different RTS morphologies. The results of this study indicate that the model shows some potential for automated RTS monitoring using medium-resolution satellite data, potentially providing a reference for large-scale permafrost degradation assessment in climate change research.

Despite MOCA-Net’s good performance, several limitations require further improvement. First, ESRGAN was used only as an image-enhancement preprocessing step. It cannot reconstruct genuine 2.5 m surface details from the original 10 m Sentinel-2 imagery, and the ESRGAN-related improvement should therefore be interpreted with caution. Second, the current approach relies on RGB optical imagery only and does not incorporate additional multispectral information or topographic variables, such as NIR/SWIR bands and DEM-derived slope and aspect, which may limit the discrimination of RTSs from spectrally similar background features and contribute to false positives in areas such as bare soil and other erosional landforms.

Future improvements should focus on multimodal integration incorporating multispectral, geological, and topographic data to reduce false positives, as well as spatiotemporal analysis for dynamic monitoring.

## Figures and Tables

**Table 1:** Evaluation metrics from eight models.

Model	Test Loss	mIoU	RTS IoU	Dice	Precision	Recall
DeepLabv3+ Xception71	0.0617	0.8503	0.7286	0.8418	0.8403	0.8458
FCN ResNet152	0.0592	0.8506	0.7284	0.8419	0.8609	0.8257
U-Net	0.0632	0.8408	0.7109	0.8300	0.8497	0.8133
HRNet	0.0604	0.8446	0.7172	0.8344	0.8627	0.8108
Vision Transformer	0.0553	0.8537	0.7336	0.8453	0.8565	0.8375
SegFormer	0.0559	0.8578	0.7423	0.8502	0.8785	0.8278
Swin + Basic Decoder	0.0585	0.8542	0.7359	0.8471	0.8355	0.8617
MOCA-Net	0.0542	0.8609	0.7473	0.8547	0.8516	0.8606

**Table 2:** Comparison of segmentation performance with and without ESRGAN preprocessing.

Model	Preprocessing	Test Loss	mIoU	RTS IoU	Dice	Precision	Recall
Swin + Basic Decoder	With ESRGAN	0.0585	0.8542	0.7359	0.8471	0.8355	0.8617
Swin + Basic Decoder	Without ESRGAN	0.0601	0.8455	0.7196	0.8334	0.8753	0.8035
MOCA-Net	With ESRGAN	0.0542	0.8609	0.7473	0.8547	0.8516	0.8606
MOCA-Net	Without ESRGAN	0.0562	0.8500	0.7275	0.8387	0.8783	0.8111

**Table 3:** Repeated-run results of MOCA-Net and the Swin + Basic Decoder across five random seeds under the ESRGAN-enhanced setting.

Model	Seed	Test Loss	mIoU	RTS IoU	Dice	Precision	Recall
Swin + Basic Decoder	42	0.0585	0.8542	0.7359	0.8471	0.8355	0.8617
Swin + Basic Decoder	62	0.0663	0.8519	0.7348	0.8466	0.8710	0.8257
Swin + Basic Decoder	82	0.0571	0.8536	0.7340	0.8458	0.8548	0.8389
Swin + Basic Decoder	102	0.0680	0.8534	0.7399	0.8501	0.8456	0.8562
Swin + Basic Decoder	122	0.0618	0.8512	0.7321	0.8445	0.8528	0.8384
Swin + Basic Decoder	Mean ± SD	0.0623 ± 0.0047	0.8529 ± 0.0013	0.7353 ± 0.0029	0.8468 ± 0.0021	0.8519 ± 0.0131	0.8442 ± 0.0146
MOCA-Net	42	0.0542	0.8609	0.7473	0.8547	0.8516	0.8606
MOCA-Net	62	0.0632	0.8604	0.7504	0.8566	0.8682	0.8470
MOCA-Net	82	0.0550	0.8594	0.7449	0.8530	0.8519	0.8555
MOCA-Net	102	0.0644	0.8589	0.7491	0.8562	0.8641	0.8502
MOCA-Net	122	0.0590	0.8579	0.7437	0.8523	0.8684	0.8385
MOCA-Net	Mean ± SD	0.0592 ± 0.0046	0.8595 ± 0.0012	0.7471 ± 0.0028	0.8546 ± 0.0019	0.8608 ± 0.0085	0.8503 ± 0.0084

**Table 4:** Statistical summary of repeated-run performance across five random seeds under the ESRGAN-enhanced setting. The 95% confidence intervals are reported for the mean values, and *p*-values are obtained from paired *t*-tests between MOCA-Net and the Swin + Basic Decoder.

Metric	Swin + Basic Decoder (Mean ± SD)	95% CI	MOCA-Net (Mean ± SD)	95% CI	Paired *p*-Value
mIoU	0.8529 ± 0.0013	[0.8513, 0.8544]	0.8595 ± 0.0012	[0.8580, 0.8610]	0.00023
RTS IoU	0.7353 ± 0.0029	[0.7317, 0.7390]	0.7471 ± 0.0028	[0.7436, 0.7505]	0.00036
Dice	0.8468 ± 0.0021	[0.8442, 0.8494]	0.8546 ± 0.0019	[0.8522, 0.8569]	0.00027

**Table 5:** Ablation experiments for MOCA-Net.

Model	Test Loss	mIoU	RTS IoU	Dice	Precision	Recall
M0: Swin + Basic Decoder	0.0585	0.8542	0.7359	0.8471	0.8355	0.8617
M1: M0 + Feature Enhancement	0.0549	0.8584	0.7424	0.8516	0.8581	0.8483
M2: Swin + Enhanced Decoder	0.0567	0.8572	0.7405	0.8501	0.8628	0.8397
M3: MOCA-Net	0.0542	0.8609	0.7473	0.8547	0.8516	0.8606

**Table 6:** Comparison of segmentation performance and computational complexity between MOCA-Net and the Swin + Basic Decoder baseline. The mIoU and RTS IoU values are taken from the main test results reported in Section 4.2, whereas inference time was benchmarked separately on an NVIDIA GeForce RTX 4050 Laptop GPU using an input size of 224 × 224 and a batch size of 1.

Model	mIoU	RTS IoU	Parameters (M)	FLOPs (G)	Inference Time (ms/Image)
Swin + Basic Decoder	0.8542	0.7359	92.594	17.793	32.962
MOCA-Net	0.8609	0.7473	111.744	89.031	38.059

## Data Availability

The Sentinel-2 imagery is freely available from the Google Earth Engine platform. The RTS inventory data supporting this study are available from the published dataset [66] at https://doi.org/10.5194/essd-14-3875-2022 (accessed on 17 May 2026). The source code for MOCA-Net is publicly available at https://github.com/qtds12321/MOCA-Net (accessed on 17 May 2026), and the pre-trained model weights are available at https://github.com/qtds12321/MOCA-Net/releases/tag/v1.0 (accessed on 17 May 2026).

## Abbreviations

The following abbreviations are used in this manuscript:

RTSs	Retrogressive Thaw Slumps
QTEC	Qinghai–Tibet Engineering Corridor
MOCA-Net	Multi-Scale Object-aware Context Attention Network
IoU	Intersection over Union
mIoU	Mean Intersection over Union
ESRGAN	Enhanced Super-Resolution Generative Adversarial Network
CNN	Convolutional Neural Network
ViT	Vision Transformer
Swin Transformer	Shifted Window Transformer
